# Supplementary material for: AI-Aided Design of Novel Targeted Covalent Inhibitors against SARS-CoV-2
Source: Biomolecules. 2022 May 25;12(6):746. doi: 10.3390/biom12060746 (PMC9220321; doi:10.3390/biom12060746)
Supplement: Supplementary file 1 [file biomolecules-12-00746-s001.zip › biomolecules-1704234-supplementary.pdf]

Supporting Materials

```
Query  3241  FSNSGSDVLYQPPQTSITSAVLQSGFRKMAFPSGKVEGCMVQVTCGTTTLNGLWLDDVVY  3300
                                     SGFRKMAFPSGKVEGCMVQVTCGTTTLNGLWLDD VY
Sbjct  1                             SGFRKMAFPSGKVEGCMVQVTCGTTTLNGLWLDDTVY  37

Query  3301  CPRHVICTSEDMLNPNYEDLLIRKSNHNFLVQAGNVQLRVIGHSMQNCVLKLKVDTANPK  3360
             CPRHVICT+EDMLNPNYEDLLIRKSNH+FLVQAGNVQLRVIGHSMQNC+L+LKVDT+NPK
Sbjct  38    CPRHVICTAEDMLNPNYEDLLIRKSNHSFLVQAGNVQLRVIGHSMQNCLLRLKVDTSNPK  97

Query  3361  TPKYKFVRIQPGQTFSVLACYNGSPSGVYQCAMRPNFTIKGSFLNGSCGSVGFNIDYDCV  3420
             TPKYKFVRIQPGQTFSVLACYNGSPSGVYQCAMRPN TIKGSFLNGSCGSVGFNIDYDCV
Sbjct  98    TPKYKFVRIQPGQTFSVLACYNGSPSGVYQCAMRPNHTIKGSFLNGSCGSVGFNIDYDCV  157

Query  3421  SFCYMHHMELPTGVHAGTDLEGNFYGPFVDRQTAQAAGTDTTITVNVLAWLYAAVINGDR  3480
             SFCYMHHMELPTGVHAGTDLEG FYGPFVDRQTAQAAGTDTTIT+NVLAWLYAAVINGDR
Sbjct  158   SFCYMHHMELPTGVHAGTDLEGKFYGPFVDRQTAQAAGTDTTITLNVLAWLYAAVINGDR  217

Query  3481  WFLNRFTTTLNDFNLVAMKYNYEPLTQDHVDILGPLSAQTGIAVLDMCASLKELLQNGMN  3540
             WFLNRFTTTLNDFNLVAMKYNYEPLTQDHVDILGPLSAQTGIAVLDMCA+LKELLQNGMN
Sbjct  218   WFLNRFTTTLNDFNLVAMKYNYEPLTQDHVDILGPLSAQTGIAVLDMCAALKELLQNGMN  277

Query  3541  GRTILGSALLEDEFTPFDVVRQCSGVTFQSAVKRTIKGTHHWLLLTILTSLLVLVQSTQW  3600
             GRTILGS +LEDEFTPFDVVRQCSGVTFQ
Sbjct  278   GRTILGSTILEDEFTPFDVVRQCSGVTFQ                                 306
```

```
WUHAN|Viral             1   SGFRKMAFPSGKVEGCMVQVTCGTTTLNGLWLDDVVYCPRHVICTSEDMLNPNYEDLLIR    60
5N5O:A|PDBID|CH..       1   SGFRKMAFPSGKVEGCMVQVTCGTTTLNGLWLDDTVYCPRHVICTAEDMLNPNYEDLLIR    60
                            **********************************.**********:**************

WUHAN|Viral            61   KSNHNFLVQAGNVQLRVIGHSMQNCVLKLKVDTANPKTPKYKFVRIQPGQTFSVLACYNG   120
5N5O:A|PDBID|CH..      61   KSNHSFLVQAGNVQLRVIGHSMQNCLLRLKVDTSNPKTPKYKFVRIQPGQTFSVLACYNG   120
                            ****.********************:*:*****:**************************

WUHAN|Viral           121   SPSGVYQCAMRPNFTIKGSFLNGSCGSVGFNIDYDCVSFCYMHHMELPTGVHAGTDLEGN   180
5N5O:A|PDBID|CH..     121   SPSGVYQCAMRPNHTIKGSFLNGSCGSVGFNIDYDCVSFCYMHHMELPTGVHAGTDLEGK   180
                            ************.**********************************************:

WUHAN|Viral           181   FYGPFVDRQTAQAAGTDTTITVNVLAWLYAAVINGDRWFLNRFTTTLNDFNLVAMKYNYE   240
5N5O:A|PDBID|CH..     181   FYGPFVDRQTAQAAGTDTTITLNVLAWLYAAVINGDRWFLNRFTTTLNDFNLVAMKYNYE   240
                            *********************:**************************************

WUHAN|Viral           241   PLTQDHVDILGPLSAQTGIAVLDMCASLKELLQNGMNGRTILGSALLEDEFTPFDVVRQC   300
5N5O:A|PDBID|CH..     241   PLTQDHVDILGPLSAQTGIAVLDMCAALKELLQNGMNGRTILGSTILEDEFTPFDVVRQC   300
                            **************************:*****************::**************

WUHAN|Viral           301   SGVTFQ                                                         306
5N5O:A|PDBID|CH..     301   SGVTFQ                                                         306
                            ******
```

Date of job execution	2020-01-28
Job identifier	A20200128674680 3381A1F0E0DB47453E0216320D052666Q (jobs are stored for 7 days)
Running time	13.8 seconds
Identical positions	294
Identity	96.078%
Similar positions	12
Program	CLUSTALO

Figure S1. Sequence alignment between SARS-CoV-2 3CL^{pro} and SARS-CoV 3CL^{pro}.

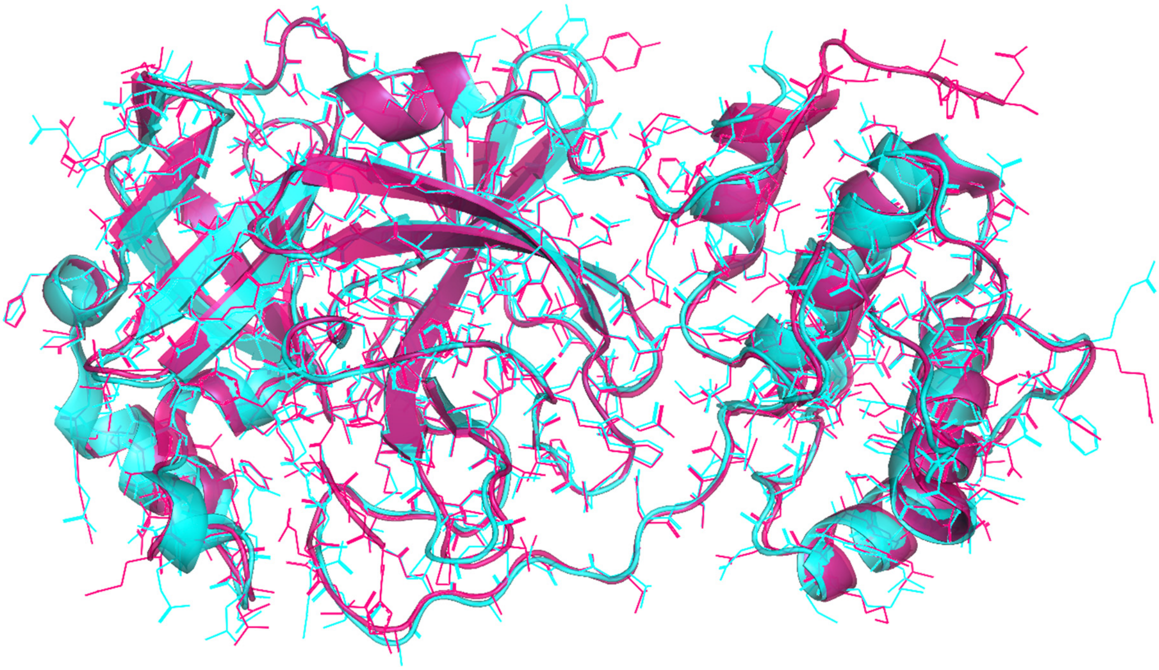

Figure S2. Structure superposition between SARS-CoV-2 3CL^{pro} (PDB ID: 6LU7 shown in magenta) and SARS-CoV 3CL^{pro} (PDB ID: 3D62 shown in cyan) with an RMSD of 0.44 Å.

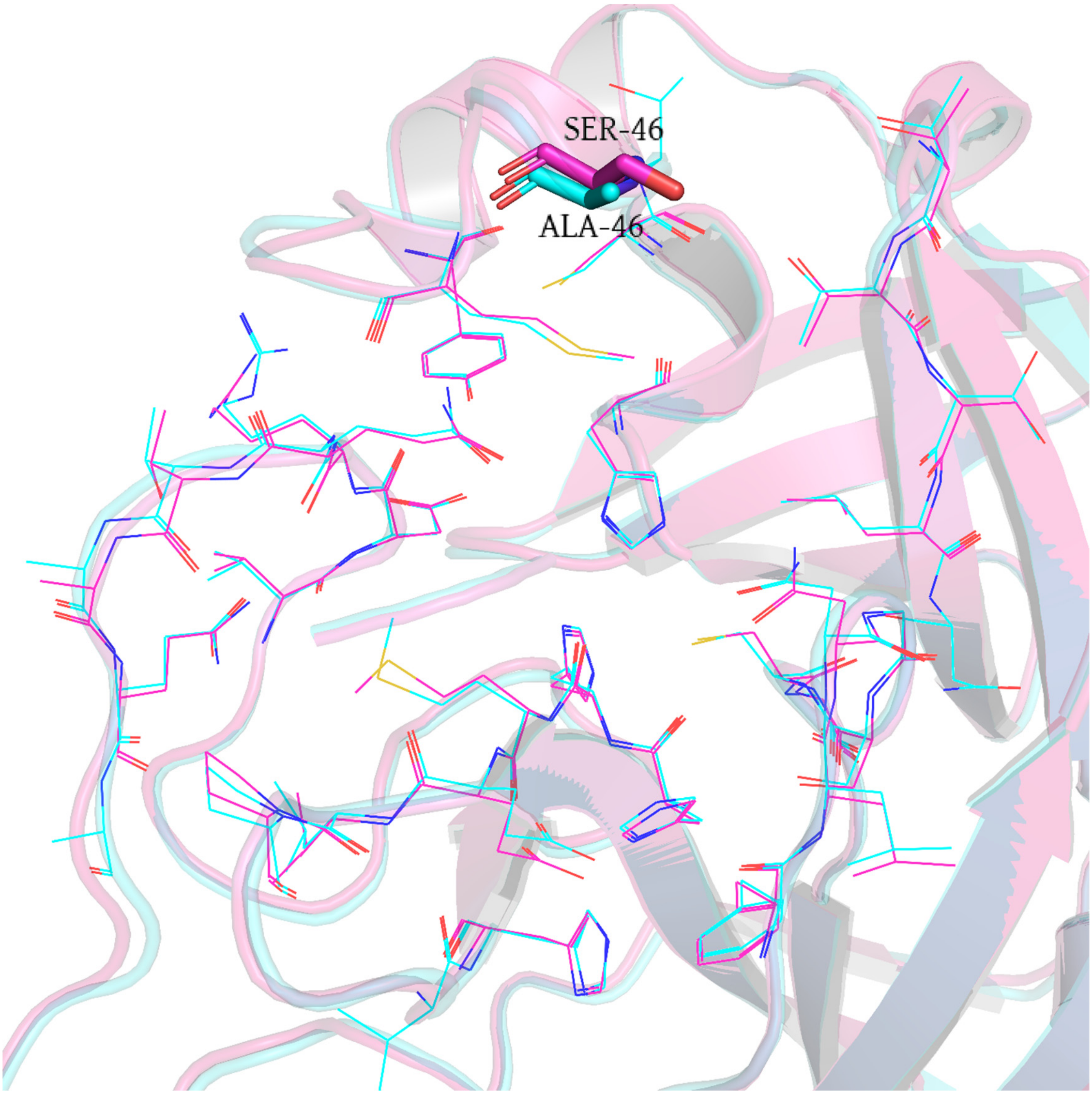

Figure S3. Substrate-binding site superimposition of SARS-CoV-2 3CL^{pro} (PDB ID: 6LU7, magenta cartoon) and SARS-CoV 3CL^{pro} (PDB ID: 2HOB, cyan cartoon). Amino acid residues in the 6Å range of the original molecular ligand N3 (SARS-CoV-2 3CL^{pro}: lines in magentas, SARS-CoV 3CL^{pro}: lines in cyan) are selected for comparative analysis, and only the residues at position 46 are different. The residue of SARS-CoV-2 3CL^{pro} at position 46 is serine (shown with sticks in magentas), while alanine in SARS-CoV 3CL^{pro} (shown with sticks in cyan).

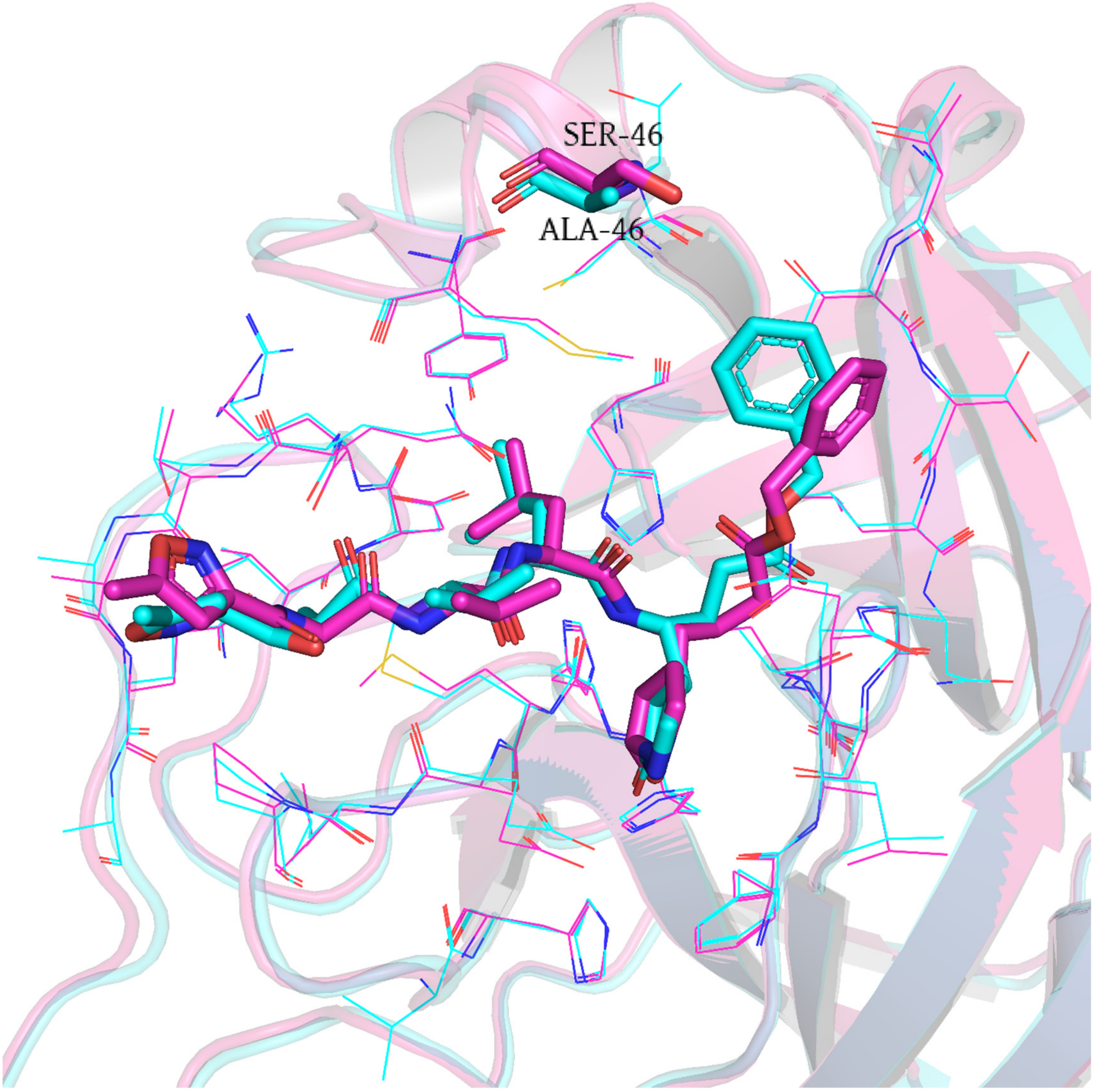

Figure S4. Comparison of the ligand conformations between SARS-CoV-2 3CL^{pro} (PDB ID:6LU7) and SARS-CoV 3CL^{pro} (PDB ID:2HOB). The ligand structure of SARS-CoV-2 3CL^{pro} is shown with sticks in magenta, while the ligand of SARS-CoV 3CL^{pro} is shown with sticks in cyan.

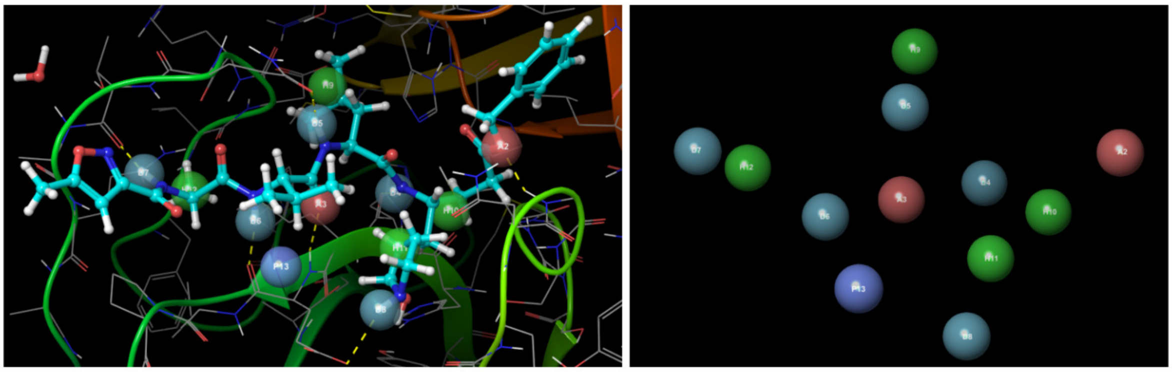

Figure S5. The pharmacophore model embedded in the *R* function. The pharmacophore consists of two hydrogen receptors (red spheres), five hydrogen donors (light blue spheres), four hydrophobic characteristics (green spheres) and a positive charge center (blue spheres).

Tables S1. Results of non-covalent and covalent docking results.

ID	docking score	cdock affinity	KabschRmsd	StraightRmsd	SMILES
1	-7.63				N1CC[C@H](C1=O)N[C@H](C(=O)C=O)Cn2nnc(c23)cccc3Oc(c4cccc5)nc(c6c45)cccc6
2	-7.743	-5.464	2.752	9.245	N1CC[C@H](C1=O)[C@H](C[C@H]2C)N(CC2)C(=O)/C=C/[C@@](C)(NC(=O)c3ccc(o3)Cl)OCc(c4)ccc(c45)n(C)c(n5)C
3	-6.802	-6.374	2.381	6.455	c1c[nH]cc(c12)nc(n2)N[C@@](/C=C/C=O)(C[C@@H](C3=O)CCN3)c4c(O)c(c(o4)C=O)-c5c(Cl)cc(F)cc5
4	-6.807	-6.102	2.491	8.626	N1CC[C@H](C1=O)C[C@H](N)\C=C\C(=O)Nc(ccc2)c(c2CCC=O)-n3nnc(c34)cccc4C[C@@H](N)CC=O
5	-8.739	-8.171	4.254	8.37	NC(=O)COC(=O)/C=C/[C@@H](N)C[C@H]1[C@H](Oc(c2cccc3)nc(c4c23)cccc4)OC(=O)[C@@]1([C@@H](C=O)N)C[C@@H](C5=O)CCN5
6	-8.772	-6.262	4.314	9.295	FCC(=O)c1ccc(o1)[C@@](N)(C[C@@H](C2=O)CCN2)\C=C\C(=O)N[C@H](C=O)[C@@H](O)Cc(c3)sc(c34)cccc4
7	-7.787				N1CC[C@H](C1=O)N[C@H](C(=O)C=O)Cc2ccc(Cl)cc2
8	-7.349				O=CC(=O)[C@@H](N)Cc1ccnc(c12)c(c(CN)cc2)C[C@@H](C3=O)CCN3
9	-8.874	-7.388	1.473	1.734	O=c1c(O)coc(c12)cc(cc2O)NC(=N/[H])\c3c(CCC)cc(cc3)N[C@](N)(/C=C/C=O)C[C@@H](C4=O)CCN4
10	-8.967	-4.159	2.393	11.36	N1CC[C@H](C1=O)C[C@@H](N)\C=C\C(=O)N(C)C(=O)[C@H](C)[C@@H](c(s2)ccc2C=O)[C@H](O)[C@@H](N)C(C)(C)c3cccc(c34)cccc4
11	-9.075	-6.923	3.952	5.132	N1CC[C@H](C1=O)C[C@@](N)(/C=C/C=O)c2c(cc(s2)C=O)-c(n3)nc(c34)cn(C)cc4N[C@](C=O)(C)c5cnc[nH]5
12	-7.256	-5.275	3.352	7.935	O=C\C=C\[C@](C)(N)[C@@H](C1=O)[C@H](OCC=O)CN1C(=O)[C@@H](c(c2)ccc(c23)n(C(=O)C)cc3)C(C)(C)c(c4)sc(c45)cccc5
13	-7.545	-7.222	2.834	4.748	N1CC[C@H](C1=O)C[C@](N)(/C=C/C=O)N(C2=O)CCCN2c(cc3O)cc(c34)occ(c4=O)N[C@H](C=O)C
14	-6.823	-5.008	1.639	4.562	s1cccc1[C@@]([C@H](N)C(=O)C=O)([C@@H](C2=O)CCN2)c3ccc(cc3)\C=C\C=O
15	-8.301				N1CC[C@H](C1=O)N[C@H](C(=O)C=O)Cc2c(C3CCC3)cc(F)cc2Cl
16	-7.663	-7.199	1.837	4.789	N1CC[C@H](C1=O)N[C@H](C(=O)C(=O)O)Cc(c2)sc(c23)cccc3
17	-6.766	-5.437	3.614	4.207	O=c(o1)oc(C)c1CN[C@](/C=C/C=O)(C[C@@H](C2=O)CCN2)c3c(ccc(=O)o3)O[C@H]4CO[C@H]([C@H]45)OCC5
18	-7.404				N1CC[C@H](C1=O)C[C@@H](C=O)N(CC=O)[C@H](C(=O)C=O)Cc2cc(N)ccc2
19	-7.632	-8.385	2.899	5.489	N1CC[C@H](C1=O)C[C@](N)(/C=C/C=O)[C@H](CC2)C[C@]2(N[C@H](C=O)[C@@H](C)O)c(cc3=O)oc(c34)cc(O)cc4O
20	-7.234	-6.597	1.873	2.615	N1CC[C@H](C1=O)N[C@H](C(=O)C(=O)O)Cc(c(c2=O)O)oc(c23)c(O)c(O)cc3O
21	-8.133	-6.518	1.907	8.581	N1CC[C@H](C1=O)C[C@@H](N)\C=C\C(=O)O[C@H]2CO[C@H]([C@H]23)O[C@](C3)(NC(C)(C)CC)N(C(=O)CC)c4ccccc4
22	-7.2	-7.392	4.657	6.777	n1nccn1-c(cs2)nc2C(=O)[C@H](C[C@@H](C3=O)CCN3)N[C@H](C=O)[C@H](O)C(=O)N[C@@H](C)\C=C\C=O
23	-7.625	-6.972	2.61	7.52	c1cccc(c12)c(=O)[nH]n(c2=O)[C@@](N)(\C=C\C(=O)N[C@H](C(=O)O)CC)C[C@@H](C3=O)C[C@@H](N3)C[C@H](N)CC
24	-7.211				O=CC(=O)[C@@H](N)Cc(nn1)n(c12)C[C@@](N)(NC2)CCC[C@@H](C3=O)CCN3
25	-8.133				N1CC[C@H](C1=O)N[C@H](C(=O)C=O)Cc2c(O)c(Cl)cc(Cl)c2Cl
26	-8.346	-8.322	3.402	8.209	C[C@H](N)C(=O)NN(CCC(=O)N)C(=O)c(c(c12)[nH]cc1)c(C[C@@](N)(/C=C/C=O)[C@@H](C3=O)CCN3)cc2C[C@@H](N)CC=O
27	-7.308				N1CC[C@H](C1=O)N[C@H](C(=O)C=O)Cc2cccs2
28	-6.845				s1cccc1C[C@@H](C(=O)C=O)NCC[C@@H](N)C[C@@H](C2=O)CCN2
29	-8.176	-7.667	1.921	2.503	CC(C)C[C@@H](B(O)O)Nc(cs1)nc1C(=O)[C@H](C)N(C(=O)[C@@H](N)CC)C(=O)/C=C/[C@@H](N)C[C@@H](C2=O)CCN2
30	-6.241				C1CC1C[C@@H](C(=O)C=O)N[C@@H](C2=O)CCN2
31	-6.502				N1CC[C@H](C1=O)N[C@H](C(=O)C=O)CC2CCCC2
32	-5.731				O=CC(=O)[C@@H](N)C[C@@H](C1=O)C[C@@H](C[C@H](N)CC)N1[C@@]2(OCC(C)C)CC[C@@H](O)CC2
33	-7.246				N1CC[C@H](C1=O)[C@H]2N[C@H](C[C@H](N)C(=O)C=O)C[C@@H]([C@@H]23)[C@H](SC)CCC3
34	-5.337				O=CC(=O)[C@@H](N)C[C@@H](C1=O)CCN1
35	-5.52	-5.461	2.191	6.963	C[C@H](N)C(=O)C(=O)NC(=O)/C=C/[C@H](N)C[C@@H](C1=O)CCN1
36	-4.847				O=CC(=O)[C@H](C)NC(=O)[C@H](CC(C)C)N[C@@H](C1=O)CCN1
37	-7.325	-5.179	1.83	2.911	O=CC(=O)[C@H](C)N(C=O)C(=O)/C=C/[C@H](N)C[C@@H](C1=O)CCN1
38	-6.459				O=CC(=O)[C@@H](N)[C@H](N)[C@@H]1[C@H]([C@@H](C2=O)CCN2)NC[C@@H](N1)N(C)C
39	-7.998	-6.302	3.796	9.2	n1cc(C)ncc1C(=O)N[C@H](C[C@@H](C2=O)CCN2)\C=C\C(=O)Oc(nc(c3c45)cccc3)c4cc(cc5)-c6ccc([N+]([O-])=O)cc6
40	-7.779	-6.513	2.76	6.048	n1ccncc1C(=O)N[C@H](\C=C\C=O)[C@]([C@@H](C2=O)CCN2)(c3ccccc3)Oc(c4cccc5)nc(c6c45)cccc6
41	-7.196	-4.571	3.056	3.748	c1ccccc1O[C@@](C)(/C=C/C=O)NC(=O)[C@@H](c2cccnn2)[C@H](C)C[C@@H](C3=O)CCN3c4nccs4
42	-7.931	-4.447	4.241	6.345	c1ccccc1\C=C\C(=O)N([C@@H](\C=C\C=O)C[C@@H](C2=O)CCN2)[C@H]([C@@H](O)C[C@H](C=O)C)c3c(F)cc(F)c(F)c3
43	-5.985				O=CC(=O)[C@H](CN)N[C@@H](C1=O)CCN1
44	-7.013	-5.197	1.304	2.97	s1cccc1C(=O)N([C@](C)(/C=C/C=O)[C@@H](C2=O)CCN2)c3c(n[nH]n3)Nc(on4)c([N+]([O-])=O)c4C
45	-8.227	-5.941	3.01	6.185	Cc1onc(C)c1-c(cco2)c2\C=C(\C=O)NC(=O)/C=C/[C@H](C[C@@H](C3=O)CCN3)NC(=O)N[C@H](C=O)C
46	-8.19	-8.722	1.71	2.131	[nH]1nncc1N([C@@H](C)\C=C\C=O)C(=O)[C@@H]([C@H](C)C[C@@H](C2=O)CCN2)c(cc3=O)oc(c34)cc(O)cc4O
47	-5.513				O=CC(=O)[C@H](C)N[C@@H](C1=O)CCN1

Several lead compounds do not have the covalent docking results as their reactive group is in an unsuitable direction to 145CYS. All RMSD values are heavy atoms based. The unity is kcal/mol

KabschRmsd: Root-mean-square deviation (RMSD) is calculated with the Kabsch algorithm (1976) doi: http://dx.doi.org/10.1107/S0567739476001873).

StraightRmsd: Root-mean-square deviation (RMSD) is calculated directly from the atomic coordinates without superimposing the atoms.

The unity for Both RMSD is Å

Table S2. 45 rules pf different chemical reactions.

[$([C;D3]([#0,#6,#7,#8])(=O)):1]-;!@[$([O;D2]-;!@[#0,#6,#1]):2]>>[1*]-[*:1].[3*]-[*:2]
[$([C;D3]([#0,#6,#7,#8])(=O)):1]-;!@[$([N;!D1;!$(N=*);!$(N-[!#6;!#16;!#0;!#1]);!$([N;R]@[C;R]=O)]):2]>>[1*]-[*:1].[5*]-[*:2]
[$([C;D3]([#0,#6,#7,#8])(=O)):1]-;!@[$([N;R;$(N(@C(=O))@[C,N,O,S])]):2]>>[1*]-[*:1].[10*]-[*:2]
[$([O;D2]-;!@[#0,#6,#1]):1]-;!@[$([C;!D1;!$(C=*)]-;!@[#6]):2]>>[3*]-[*:1].[4*]-[*:2]
[$([O;D2]-;!@[#0,#6,#1]):1]-;!@[$([C;$(C(-;@[C,N,O,S])-;@[N,O,S])]):2]>>[3*]-[*:1].[13*]-[*:2]
[$([O;D2]-;!@[#0,#6,#1]):1]-;!@[$([c;$(c(:[c,n,o,s]):[n,o,s])]):2]>>[3*]-[*:1].[14*]-[*:2]
[$([O;D2]-;!@[#0,#6,#1]):1]-;!@[$([C;$(C(-;@C)-;@C)]):2]>>[3*]-[*:1].[15*]-[*:2]
[$([O;D2]-;!@[#0,#6,#1]):1]-;!@[$([c;$(c(:c):c)]):2]>>[3*]-[*:1].[16*]-[*:2]
[$([C;!D1;!$(C=*)]-;!@[#6]):1]-;!@[$([N;!D1;!$(N=*);!$(N-[!#6;!#16;!#0;!#1]);!$([N;R]@[C;R]=O)]):2]>>[4*]-[*:1].[5*]-[*:2]
[$([C;!D1;!$(C=*)]-;!@[#6]):1]-;!@[$([S;D2](-;!@[#0,#6])):2]>>[4*]-[*:1].[11*]-[*:2]
[$([N;!D1;!$(N=*);!$(N-[!#6;!#16;!#0;!#1]);!$([N;R]@[C;R]=O)]):1]-;!@[$([S;D4]([#6,#0])(=O)(=O)):2]>>[5*]-[*:1].[12*]-[*:2]
[$([N;!D1;!$(N=*);!$(N-[!#6;!#16;!#0;!#1]);!$([N;R]@[C;R]=O)]):1]-;!@[$([c;$(c(:[c,n,o,s]):[n,o,s])]):2]>>[5*]-[*:1].[14*]-[*:2]
[$([N;!D1;!$(N=*);!$(N-[!#6;!#16;!#0;!#1]);!$([N;R]@[C;R]=O)]):1]-;!@[$([c;$(c(:c):c)]):2]>>[5*]-[*:1].[16*]-[*:2]
[$([N;!D1;!$(N=*);!$(N-[!#6;!#16;!#0;!#1]);!$([N;R]@[C;R]=O)]):1]-;!@[$([C;$(C(-;@[C,N,O,S])-;@[N,O,S])]):2]>>[5*]-[*:1].[13*]-[*:2]
[$([N;!D1;!$(N=*);!$(N-[!#6;!#16;!#0;!#1]);!$([N;R]@[C;R]=O)]):1]-;!@[$([C;$(C(-;@C)-;@C)]):2]>>[5*]-[*:1].[15*]-[*:2]
[$([C;D3;!R](=O)-;!@[#0,#6,#7,#8]):1]-;!@[$([C;$(C(-;@[C,N,O,S])-;@[N,O,S])]):2]>>[6*]-[*:1].[13*]-[*:2]
[$([C;D3;!R](=O)-;!@[#0,#6,#7,#8]):1]-;!@[$([c;$(c(:[c,n,o,s]):[n,o,s])]):2]>>[6*]-[*:1].[14*]-[*:2]
[$([C;D3;!R](=O)-;!@[#0,#6,#7,#8]):1]-;!@[$([C;$(C(-;@C)-;@C)]):2]>>[6*]-[*:1].[15*]-[*:2]
[$([C;D3;!R](=O)-;!@[#0,#6,#7,#8]):1]-;!@[$([c;$(c(:c):c)]):2]>>[6*]-[*:1].[16*]-[*:2]
[$([C;!R;!D1;!$(C!-*)]):1]-;!@[$([n;+0;$(n(:[c,n,o,s]):[c,n,o,s])]):2]>>[8*]-[*:1].[9*]-[*:2]
[$([C;!R;!D1;!$(C!-*)]):1]-;!@[$([N;R;$(N(@C(=O))@[C,N,O,S])]):2]>>[8*]-[*:1].[10*]-[*:2]
[$([C;!R;!D1;!$(C!-*)]):1]-;!@[$([C;$(C(-;@[C,N,O,S])-;@[N,O,S])]):2]>>[8*]-[*:1].[13*]-[*:2]
[$([C;!R;!D1;!$(C!-*)]):1]-;!@[$([c;$(c(:[c,n,o,s]):[n,o,s])]):2]>>[8*]-[*:1].[14*]-[*:2]
[$([C;!R;!D1;!$(C!-*)]):1]-;!@[$([C;$(C(-;@C)-;@C)]):2]>>[8*]-[*:1].[15*]-[*:2]
[$([C;!R;!D1;!$(C!-*)]):1]-;!@[$([c;$(c(:c):c)]):2]>>[8*]-[*:1].[16*]-[*:2]
[$([n;+0;$(n(:[c,n,o,s]):[c,n,o,s])]):1]-;!@[$([C;$(C(-;@[C,N,O,S])-;@[N,O,S])]):2]>>[9*]-[*:1].[13*]-[*:2]
[$([n;+0;$(n(:[c,n,o,s]):[c,n,o,s])]):1]-;!@[$([c;$(c(:[c,n,o,s]):[n,o,s])]):2]>>[9*]-[*:1].[14*]-[*:2]
[$([n;+0;$(n(:[c,n,o,s]):[c,n,o,s])]):1]-;!@[$([C;$(C(-;@C)-;@C)]):2]>>[9*]-[*:1].[15*]-[*:2]
[$([n;+0;$(n(:[c,n,o,s]):[c,n,o,s])]):1]-;!@[$([c;$(c(:c):c)]):2]>>[9*]-[*:1].[16*]-[*:2]
[$([N;R;$(N(@C(=O))@[C,N,O,S])]):1]-;!@[$([C;$(C(-;@[C,N,O,S])-;@[N,O,S])]):2]>>[10*]-[*:1].[13*]-[*:2]
[$([N;R;$(N(@C(=O))@[C,N,O,S])]):1]-;!@[$([c;$(c(:[c,n,o,s]):[n,o,s])]):2]>>[10*]-[*:1].[14*]-[*:2]
[$([N;R;$(N(@C(=O))@[C,N,O,S])]):1]-;!@[$([C;$(C(-;@C)-;@C)]):2]>>[10*]-[*:1].[15*]-[*:2]
[$([N;R;$(N(@C(=O))@[C,N,O,S])]):1]-;!@[$([c;$(c(:c):c)]):2]>>[10*]-[*:1].[16*]-[*:2]
[$([S;D2](-;!@[#0,#6])):1]-;!@[$([C;$(C(-;@[C,N,O,S])-;@[N,O,S])]):2]>>[11*]-[*:1].[13*]-[*:2]
[$([S;D2](-;!@[#0,#6])):1]-;!@[$([c;$(c(:[c,n,o,s]):[n,o,s])]):2]>>[11*]-[*:1].[14*]-[*:2]
[$([S;D2](-;!@[#0,#6])):1]-;!@[$([C;$(C(-;@C)-;@C)]):2]>>[11*]-[*:1].[15*]-[*:2]
[$([S;D2](-;!@[#0,#6])):1]-;!@[$([c;$(c(:c):c)]):2]>>[11*]-[*:1].[16*]-[*:2]
[$([C;$(C(-;@[C,N,O,S])-;@[N,O,S])]):1]-;!@[$([c;$(c(:[c,n,o,s]):[n,o,s])]):2]>>[13*]-[*:1].[14*]-[*:2]
[$([C;$(C(-;@[C,N,O,S])-;@[N,O,S])]):1]-;!@[$([C;$(C(-;@C)-;@C)]):2]>>[13*]-[*:1].[15*]-[*:2]
[$([C;$(C(-;@[C,N,O,S])-;@[N,O,S])]):1]-;!@[$([c;$(c(:c):c)]):2]>>[13*]-[*:1].[16*]-[*:2]

[$([c;$(c(:[c,n,o,s]):[n,o,s])]):1]-;!@[$([c;$(c(:[c,n,o,s]):[n,o,s])]):2]>>[14*]-[*:1].[14*]-[*:2]
[$([c;$(c(:[c,n,o,s]):[n,o,s])]):1]-;!@[$([C;$(C(-;@C)-;@C)]):2]>>[14*]-[*:1].[15*]-[*:2]
[$([c;$(c(:[c,n,o,s]):[n,o,s])]):1]-;!@[$([c;$(c(:c):c)]):2]>>[14*]-[*:1].[16*]-[*:2]
[$([C;$(C(-;@C)-;@C)]):1]-;!@[$([c;$(c(:c):c)]):2]>>[15*]-[*:1].[16*]-[*:2]
[$([c;$(c(:c):c)]):1]-;!@[$([c;$(c(:c):c)]):2]>>[16*]-[*:1].[16*]-[*:2]

Note, a reaction definition is based on the SMILES arbitrary target specification (SMARTS), which is a chemical language for describing molecular patterns. We also use the same reaction type for adding fragments, except with the reverse reaction.

Table S3. Five clusters for the generative 47 leads

Cluster	Name	Structure	SMILES	Canvas Mean Shape Similarity
1	25	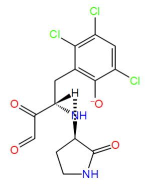	N1CC[C@H](C1=O)N[C@H](C(=O)C=O)Cc2c([O-])c(Cl)cc(Cl)c2Cl	0.001
	27	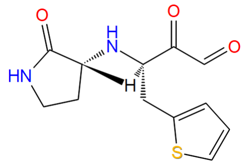	N1CC[C@H](C1=O)N[C@H](C(=O)C=O)Cc2cccs2	0.001
	7	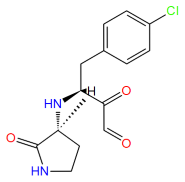	N1CC[C@H](C1=O)N[C@H](C(=O)C=O)Cc2ccc(Cl)cc2	0.001
	31	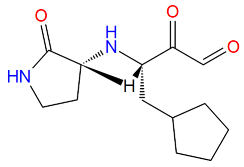	N1CC[C@H](C1=O)N[C@H](C(=O)C=O)CC2CCCC2	0.001
	47	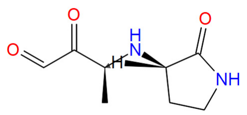	O=CC(=O)[C@H](C)N[C@@H](C1=O)CCN1	0.001

	30	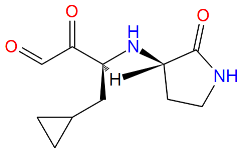	C1CC1C[C@@H](C(=O)C=O)N[C@@H](C2=O)CCN2	0.001
	43	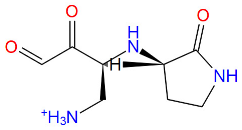	O=CC(=O)[C@H](C[NH3+])N[C@@H](C1=O)CCN1	0.001
2	36	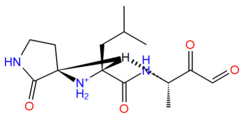	O=CC(=O)[C@H](C)NC(=O)[C@H](CC(C)C)[NH2+][C@@H](C1=O)CCN1	0.002
	15	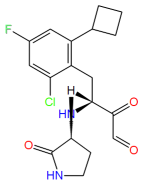	N1CC[C@H](C1=O)N[C@H](C(=O)C=O)Cc2c(C3CCC3)cc(F)cc2Cl	0.002
	34	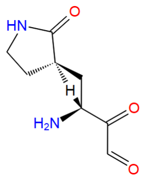	O=CC(=O)[C@@H](N)C[C@@H](C1=O)CCN1	0.002
3	32	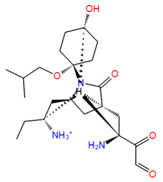	O=CC(=O)[C@@H](N)C[C@@H](C1=O)C[C@@H](C[C@H]([NH3+])CC)N1[C@@]2(OCC(C)C)CC[C@@H](O)CC2	0.003
	8	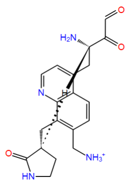	O=CC(=O)[C@@H](N)Cc1ccnc(c12)c(c(C[NH3+])cc2)C[C@@H](C3=O)CCN3	0.003
	1	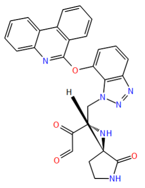	N1CC[C@H](C1=O)N[C@H](C(=O)C=O)Cn2nnc(c23)cccc3Oc(c4cccc5)nc(c6c45)cccc6	0.003

	28	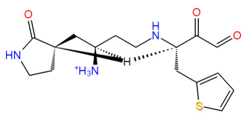	s1cccc1C[C@@H](C(=O)C=O)NCC[C@@H]([NH3+])C[C@@H](C2=O)CCN2	0.003
	18	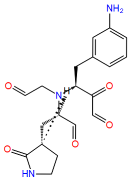	N1CC[C@H](C1=O)C[C@@H](C=O)N(CC=O)[C@H](C(=O)C=O)Cc2cc(N)ccc2	0.003
	24	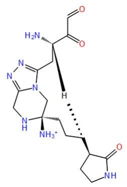	O=CC(=O)[C@@H](N)Cc(nn1)n(c12)C[C@@]([NH3+])(NC2)CCC[C@@H](C3=O)CCN3	0.003
	14	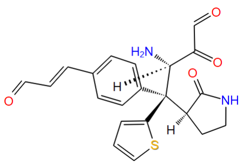	s1cccc1[C@@]([C@H](N)C(=O)C=O)([C@@H](C2=O)CCN2)c3ccc(cc3)\C=C\C=O	0.003
	33	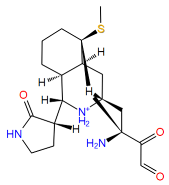	N1CC[C@H](C1=O)[C@H]2[NH2+][C@H](C[C@H](N)C(=O)C=O)C[C@@H]([C@@H]23)[C@H](SC)CCC3	0.003
	37	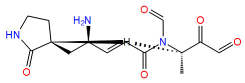	O=CC(=O)[C@H](C)N(C=O)C(=O)/C=C/[C@H](N)C[C@@H](C1=O)CCN1	0.003
	16	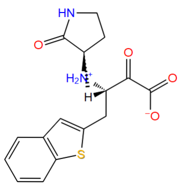	N1CC[C@H](C1=O)[NH2+][C@H](C(=O)C([O-])=O)Cc(c2)sc(c23)cccc3	0.003
	35	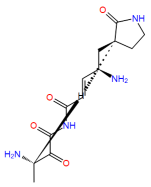	C[C@H](N)C(=O)C(=O)NC(=O)/C=C/[C@H](N)C[C@@H](C1=O)CCN1	0.003

	38	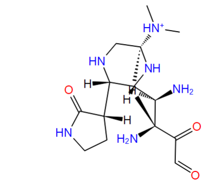	O=CC(=O)[C@@H](N)[C@H](N)[C@@H]1[C@H]([C@@H](C2=O)CCN2)NC[C@@H](N1)[NH+](C)C	0.003
4	44	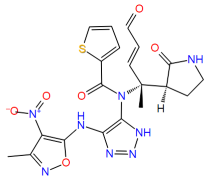	s1cccc1C(=O)N([C@](C)(/C=C/C=O)[C@@H](C2=O)CCN2)c([nH]nn3)c3Nc(on4)c([N+]([O-])=O)c4C	0.004
	17	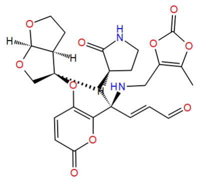	O=c(o1)oc(C)c1CN[C@](/C=C/C=O)(C[C@@H](C2=O)CCN2)c3c(ccc(=O)o3)O[C@H]4CO[C@H]([C@H]45)OCC5	0.004
	3	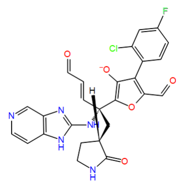	c1nccc(c12)[nH]c(n2)N[C@@](/C=C/C=O)(C[C@@H](C3=O)CCN3)c4c([O-])c(c(o4)C=O)-c5c(Cl)cc(F)cc5	0.004
	42	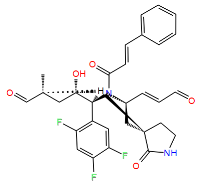	c1ccccc1\C=C\C(=O)N([C@@H](\C=C\C=O)C[C@@H](C2=O)CCN2)[C@H]([C@@H](O)C[C@H](C=O)C)c3c(F)cc(F)c(F)c3	0.004
	22	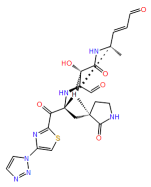	n1nccn1-c(cs2)nc2C(=O)[C@H](C[C@@H](C3=O)CCN3)N[C@H](C=O)[C@H](O)C(=O)N[C@@H](C)\C=C\C=O	0.004
	40	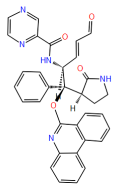	n1ccncc1C(=O)N[C@H](\C=C\C=O)[C@]([C@@H](C2=O)CCN2)(c3ccccc3)Oc(c4cccc5)nc(c6c45)cccc6	0.004
	20	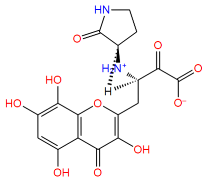	N1CC[C@H](C1=O)[NH2+][C@H](C(=O)C([O-])=O)Cc(c(c2=O)O)oc(c23)c(O)c(O)cc3O	0.004

5	2	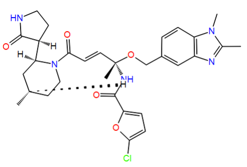	N1CC[C@H](C1=O)[C@H](C[C@H]2C)N(CC2)C(=O)/C=C/[C@@](C)(NC(=O)c3ccc(o3)Cl)OCc(c4)ccc(c45)n(C)c(n5)C	0.005
	12	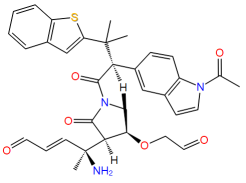	O=C\C=C\[C@](C)(N)[C@@H](C1=O)[C@H](OCC=O)CN1C(=O)[C@@H](c(c2)ccc(c23)n(C(=O)C)cc3)C(C)(C)c(c4)sc(c45)cccc5	0.005
	11	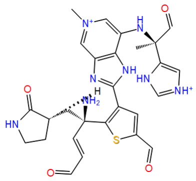	N1CC[C@H](C1=O)C[C@@](N)(/C=C/C=O)c2c(cc(s2)C=O)-c([nH]3)nc(c34)c[n+](C)cc4N[C@](C=O)(C)c5c[nH+]c[nH]5	0.005
	45	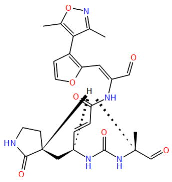	Cc1onc(C)c1-c(cco2)c2\C=C(\C=O)NC(=O)/C=C/[C@H](C[C@@H](C3=O)CCN3)NC(=O)N[C@H](C=O)C	0.005
	39	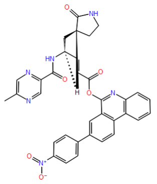	n1cc(C)ncc1C(=O)N[C@H](C[C@@H](C2=O)CCN2)\C=C\C(=O)Oc(nc(c3c45)cccc3)c4cc(cc5)-c6ccc([N+]([O-])=O)cc6	0.005
	5	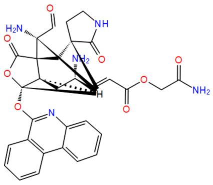	NC(=O)COC(=O)/C=C/[C@@H](N)C[C@H]1[C@H](Oc(c2cccc3)nc(c4c23)cccc4)OC(=O)[C@@]1([C@@H](C=O)N)C[C@@H](C5=O)CCN5	0.005
	46	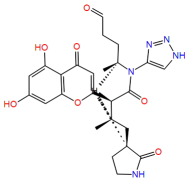	n1n[nH]cc1N([C@@H](C)CCC=O)C(=O)[C@@H]([C@H](C)C[C@@H](C2=O)CCN2)c(cc3=O)oc(c34)cc(O)cc4O	0.005

	10	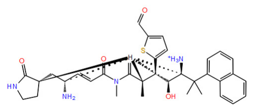	N1CC[C@H](C1=O)C[C@@H](N)\C=C\C(=O)N(C)C(=O)[C@H](C)[C@@H](c(s2)ccc2C=O)[C@H](O)[C@@H]([NH3+])C(C)(C)c3cccc(c34)cccc4	0.005
	21	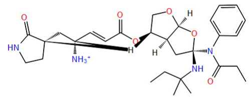	N1CC[C@H](C1=O)C[C@@H]([NH3+])\C=C\C(=O)O[C@H]2CO[C@H]([C@H]23)O[C@](C3)(NC(C)(C)CC)N(C(=O)CC)c4ccccc4	0.005
	9	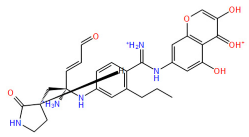	[OH+]=c1c(O)coc(c12)cc(cc2O)NC(=[NH2+])c3c(CCC)cc(cc3)N[C@]([NH3+])(/C=C/C=O)C[C@@H](C4=O)CCN4	0.005
	13	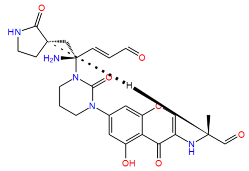	N1CC[C@H](C1=O)C[C@](N)(/C=C/C=O)N(C2=O)CCCN2c(cc3O)cc(c34)occ(c4=O)N[C@H](C=O)C	0.005
	23	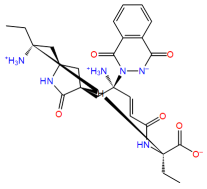	CC[C@@H](C([O-])=O)NC(=O)/C=C/[C@]([NH3+])(n([n-]c1=O)c(=O)c(c12)cccc2)C[C@@H](C3=O)C[C@@H](N3)C[C@H]([NH3+])CC	0.005
	29	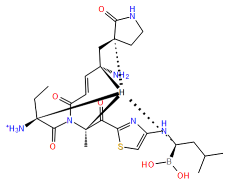	CC(C)C[C@@H](B(O)O)Nc(cs1)nc1C(=O)[C@H](C)N(C(=O)[C@@H]([NH3+])CC)C(=O)/C=C/[C@@H](N)C[C@@H](C2=O)CCN2	0.005
	19	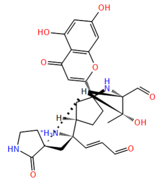	N1CC[C@H](C1=O)C[C@]([NH3+])(/C=C/C=O)[C@H](CC2)C[C@]2(N[C@H](C=O)[C@@H](C)O)c(cc3=O)oc(c34)cc(O)cc4O	0.005
	26	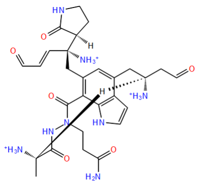	C[C@H]([NH3+])C(=O)NN(CCC(=O)N)C(=O)c(c(c12)[nH]cc1)c(C[C@@]([NH3+])(/C=C/C=O)[C@@H](C3=O)CCN3)cc2C[C@@H]([NH3+])CC=O	0.005

	4	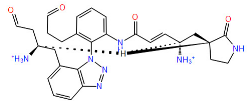	N1CC[C@H](C1=O)C[C@H]([NH3+])\C=C\C(=O)Nc(ccc2)c(c2CCC=O)-n3nnc(c34)cccc4C[C@@H]([NH3+])CC=O	0.005
	41	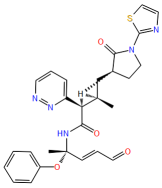	c1ccccc1O[C@@](C)(/C=C/C=O)NC(=O)[C@@H](c2cccnn2)[C@H](C)C[C@@H](C3=O)CCN3c4nccs4	0.005
	6	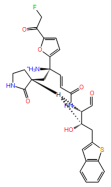	FCC(=O)c1ccc(o1)[C@@]([NH3+])(C[C@@H](C2=O)CCN2)\C=C\C(=O)N[C@H](C=O)[C@@H](O)Cc(c3)sc(c34)cccc4	0.005
